# Health and historical levels of freedom

**DOI:** 10.1186/1744-8603-6-11

**Published:** 2010-05-29

**Authors:** Samantha L Biggs, Evelyn M Dell, Vanessa L Dixon, Michel R Joffres, Chris Beyrer, Kumanan Wilson, James J Orbinski, Mills J Edward

**Affiliations:** 1 Faculty of Health Sciences, Simon Fraser University, Burnaby, Canada; 2 Johns Hopkins Bloomberg School of Public Health, Johns Hopkins University, Baltimore, USA; 3 Ottawa Hospital Research Institute, Ottawa, Canada; 4 St. Michael's Hospital, University of Toronto, Toronto, Canada; 5 Faculty of Health Sciences, University of Ottawa, Ottawa, Canada

## Abstract

**Background:**

The link between political freedom and health is unclear. We aimed to determine the association by exploring the relationship of historical and cumulative freedom levels with important health outcomes.

**Methods:**

We obtained countrywide health indicators for life expectancy, infant mortality, maternal mortality ratio, % low birth weight babies, Gini coefficient (a measure of wealth inequality) and various markers of freedom based on political rights and civil liberties. We applied multivariable logistic regression to examine the association between health indicators and within-country years of freedom as determined by Freedom House rankings.

**Results:**

The total proportion of free years from 1972-2005, the duration of current freedom level, and the Gini coefficient show independent positive associations with health indicators, which remain after the adjustment for national wealth, total government expenditure, and spending on health. Countries identified as having high total proportion of free years demonstrated significantly better health outcomes than countries with low levels of freedom (life expectancy, Odds Ratio [OR] 7.2, 95% Confidence Interval [CI], 2.3-22.6, infant mortality OR 19.6, 95% CI, 5.6-67.7, maternal mortality ratio, OR 24.3, 95% CI, 6.2-94.9, and % low birth weight babies OR 3.8, 95% CI, 1.4-10.8). This was also the case for infant mortality (OR 3.4, 95% CI, 1.0-8.4), maternal mortality ratio (OR 4.0, 95% CI, 1.2-12.8), and % low birth weight babies (OR 2.6, 95% CI, 1.0-6.6) among countries considered as having medium levels of freedom.

**Interpretation:**

We found strong associations between country-level freedom and important health outcomes. The cumulative level of freedom over time shows stronger associations with all health indicators than the duration of current freedom level.

## Background

Although the link between politics and health is often discussed,[1,2] few studies have determined the specific influence of national political rights and civil liberties on the health of individuals and populations living under them. With various organizations now providing data on political and health indicators from all over the world, large-scale global comparisons are now possible[3].

In this analysis, we explore the relationship of historical and cumulative freedom levels, based on political rights and civil liberties, using various health indicators. Previously, Franco and colleagues used freedom rankings from the Freedom House as a proxy for democracy and explored the relationship between democracy and health[4]. The authors found that higher levels of democracy were associated with better health outcomes. However, since they compared Freedom House ratings to health indicators for one year, the cross-sectional nature of the study limited the analysis of their results. For example, a country that transitioned from 'not free' to 'free' in 1998 was rated the same as a country that had been free for decades. Thus, their results did not account for the effects of recent political transition or the cumulative effect of political systems on health over time.

We expand on this issue by examining the recency of political transition and the cumulative level of freedom. In this way, we aim to determine how a country's historical level of freedom since 1972, over roughly two human generations, influences its present day health status.

## Methods

We created a database of 181 countries (in existence as of 2005) for life expectancy, infant mortality, maternal mortality ratio, % low birth weight babies, Gini coefficient (a measure of wealth distribution), total government expenditure (USD), Gross National Income/capita, % total GDP spent on health, and historical level of freedom. Data are collected from various sources,[5-13] identified in Table 1, and relate to the year 2007. Where data for 2007 was unavailable, the last observation was carried forward.

**Table 1:** Data sources for health indicators and confounders

Variable	Data Source
life expectancy	World Bank [5]
infant mortality	World Bank [5]
maternal mortality ratio	WHO [6]
% low birth weight babies	WHO,[7] World Bank [5]
Gini coefficient	Green, E.,[8] Vision of Humanity,[9] CIA,[10] World Bank [11]
total gov't expenditure	CIA [12]
GNI/capita	WHO [13]
% total GDP spent on health	World Bank [5]
freedom data	Freedom House [3]

Historical levels of freedom are measured using Freedom House ratings from 1972-2005, based on the availability of Freedom House data. Freedom House uses key metrics, that relate to political rights and civil liberties to rate countries as free, partially free, or not free for each year[3]. Political rights considered in the rating are electoral process, political pluralism and participation, and functioning of government. Civil liberties include freedom of expression and belief, associational and organizational rights, rule of law, and personal autonomy and individual rights. Methods for rating countries are described in detail elsewhere[14]. The freedom ratings are manipulated to determine the *total proportion of free years from 1972-2005 (TPFY)*, calculated as:

Here, the years of missing data are omitted from the calculation altogether, whereas 'Not Free' years are included in the denonimator. Thus, 'Not Free' years reduce the overall value of the TPFY and years of missing data do not change the TPFY. The *duration of free status (DFS)*, calculated for all countries with 'Free' status as of 2005. Countries that achieved 'Free' status in 2005 were assigned a score of 1, in 2004 were assigned a score of 2, etc. Countries scored a maximum of 34 if they were 'Free' from 1972-2005; The *duration of partially free status (DPFS)*, calculated for all countries with 'Partially Free' status as of 2005. Countries that achieved 'Partially Free' status in 2005 were assigned a score of 1, in 2004 were assigned a score of 2, etc. Countries scored a maximum of 34 if they were 'Partially Free' from 1972-2005; The *duration of not free status (DNFS)*, calculated for all countries with 'Not Free' status as of 2005. Countries that achieved 'Not Free' status in 2005 were assigned a score of 1, in 2004 were assigned a score of 2, etc. Countries scored a maximum of 34 if they were 'Not Free' from 1972-2005.

The TPFY effectively measures the cumulative level of freedom over time. The DFS, DPFS, and DNFS measure the recency of political transition. Each country has only one of DFS, DPFS, or DNFS based on their Freedom House ranking as of 2005. Once the normality of all variables was confirmed, descriptive statistics were calculated for each variable. All health and freedom variables were split into tertiles, where the lower 33% of observations are labelled as 'low', the middle 33% as 'medium', and the upper 33% as 'high'. The Gini coefficient is also split into tertiles. Despite a lower Gini signifying greater equality, for simplicity, the tertiles are labelled such that the upper 33% of observations are labelled as 'low', the middle 33% as 'medium', and the lower 33% as 'high.'

### Analysis

For health and freedom variables, we performed unadjusted and adjusted multivariable logistic regression to control for the effects of wealth (measured as per capita gross national product), level of inequality (measured with the Gini coefficient), and size of the public sector (measured as total government expenditure and percentage of GDP spent on health). For the Gini coefficient, we performed multivariable logistic regression to control for the effects of wealth (measured as per capita gross national product), size of the public sector (measured as total government expenditure and percentage of GDP spent on health), and the total proportion of free years. In the multivariate analysis, countries missing data for health indicators or control variables were excluded. Of all 181 countries considered, the unadjusted life expectancy analysis included 176 (adjusted, n = 129), unadjusted infant mortality analysis included 181 (adjusted, n = 133), unadjusted maternal mortality ratio analysis included 166 (adjusted, n = 128), and unadjusted low birth weight analysis included 173 (adjusted, n = 130) based on the availability of data. Data are presented as Odds Ratios [ORs] with 95% Confidence Intervals [CI]. All p-values are two-sided and exact. We considered a p-value of < 0.05 as statistically significant.

## Results

Our sample of 181 countries represents 98.5% of the world's population and includes 94.3% of the states recognized by the United Nations. As of 2005, 44% of the countries were considered free, 31.5% partially free, and 24% not free. Within populations exposed to lack of freedom, 17.1% and 36.2% live in a partially free and not free country, respectively. We obtained data on life expectancy for 176 countries, infant mortality for 181 countries, maternal mortality ratio for 166 countries, % low birth weight babies for 173 countries, Gini coefficient for 156 countries, total government expenditure for 173 countries, GNI/capita for 162 countries, and % of total GDP spent on health for 180 countries.

**Figure 1:**
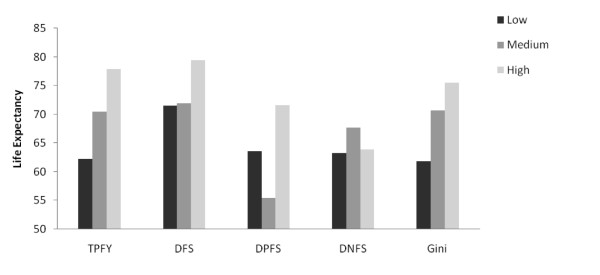
**Median life expectancy by total proportion of free years (TPFY), duration of free status (DFS), duration of partially free status (DPFS), duration of not free status (DNFS), and Gini coefficient**.

**Figure 2:**
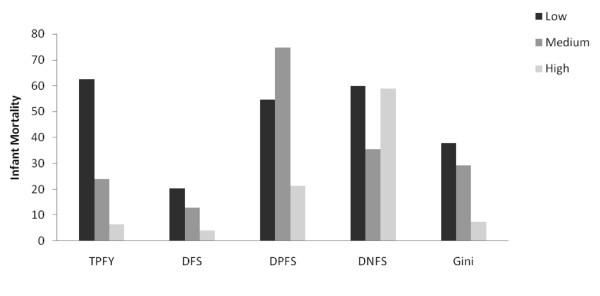
**Median infant mortality by total proportion of free years (TPFY), duration of free status (DFS), duration of partially free status (DPFS), duration of not free status (DNFS), and Gini coefficient**.

**Figure 3:**
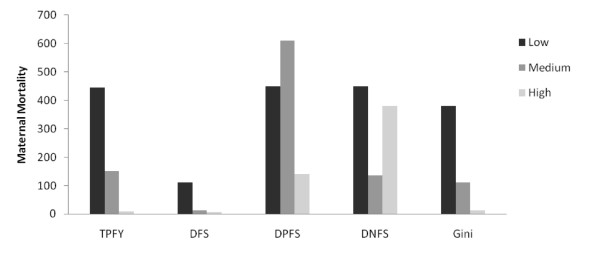
**Median maternal mortality by total proportion of free years (TPFY), duration of free status (DFS), duration of partially free status (DPFS), duration of not free status (DNFS), and Gini coefficient**.

**Figure 4:**
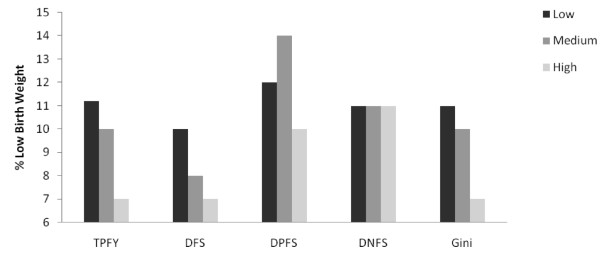
**Median percent low birth weight by total proportion of free years (TPFY), duration of free status (DFS), duration of partially free status (DPFS), duration of not free status (DNFS), and Gini coefficient**.

Health indicators were related to historical levels of freedom and the Gini coefficient (Figures 1,2,3,4). Increasing TPFY from low to high was associated with improvements in all health outcomes. The high DFS category, countries that have been free for more than 30 years, consistently had the best health outcomes. Health outcomes were worst in the medium DPFS category, countries that have been partially free for 7-15 years.

After splitting the health and freedom variables into tertiles (Table 2), the logistic regression analysis was performed. Health indicators were related to historical levels of freedom and Gini coefficient. After adjusting in the logistic regression analysis, the associations remained but fewer relationships were statistically significant (Table 3). This suggests that the effects of wealth, level of inequality, and size of the public sector have meaningful influences on health outcomes. Except for DPFS, the relationships were more often statistically significant when the category of the freedom rating is modelled from low to high.

**Table 2:** Distribution of selected variables by tertile*

		Median		
Variable	All	Low	Med	High
Life expectancy	71.0	53.8	71.0	78.3
Infant mortality	21.8	5.1	21.6	77.8
Maternal mortality ratio	130.0	8.0	130.0	700.0
% low birth weight infants	10.0	6.0	9.0	15.0
GINI	0.4	0.3	0.4	0.5
Total proportion of free years	1.0	0.3	1.0	2.0
Duration of Free Status (years)†	17.0	6.0	16.0	34.0
Duration of Partially Free Status (years)‡	11.0	3.0	11.0	23.0
Duration of Not Free Status (years)¥	14.0	6.0	14.0	34.0

* Except where otherwise indicated, sample size is 181

† Includes Free states as of 2005, n = 80

‡ Includes Partially Free states as of 2005, n = 57

¥ Includes Not Free states as of 2005, n = 44

**Table 3:** Relationship between freedom status and health status by tertile

					OR (and 95% CI)				
					
		Life expectancy		Infant mortality		Maternal mortality ratio		% low birth weight infants	
					
Variable		Unadjusted	Adjusted*	Unadjusted	Adjusted*	Unadjusted	Adjusted*	Unadjusted	Adjusted*
Total proportion of free years§	Low vs. High	12.0 (5.5-26.0)	7.2 (2.3-22.6)	17.5 (7.9-38.6)	19.6 (5.6-67.7)	25.5 (10.6-61.5)	24.3 (6.2-94.9)	4.7 (2.3-9.7)	3.8 (1.4-10.8)
	Med vs. High	5.1 (2.4-10.7)	2.1 (0.7-6.0)	6.2 (3.0-13.0)	3.0 (1.0-8.4)	9.2 (4.0-21.2)	4.0 (1.2-12.8)	3.8 (1.9-7.8)	2.6 (1.0-6.6)
Duration of Free Status (years)†	Low vs. High	21.4 (5.1-90.4)	6.6 (0.8-55.3)	29.1 (5.7-147.6)	3.6 (0.4-30.7)	18.3 (4.3-78.4)	1.9 (0.2-16.7)	4.1 (1.4-12.3)	2.4 (0.5-12.6)
	Med vs. High	10.8 (2.6-45.0)	3.1 (0.3-31.8)	13.7 (2.8-67.9)	1.2 (0.1-12.8)	3.8 (0.8-17.6)	0.4 (0.03-5.3)	2.2 (0.8-6.4)	1.6 (0.3-8.3)
Duration of Partially Free Status (years)‡	Low vs. High	3.7 (1.0-13.0)	4.8 (0.9-25.3)	3.9 (1.1-14.0)	6.1 (1.0-36.8)	3.6 (1.0-13.6)	3.4 (0.6-19.1)	1.5 (0.4-5.6)	1.3 (0.3-6.3)
	Med vs. High	3.67 (1.1-12.6)	5.1 (1.1-24.3)	4.599 (1.3-16.5)	11.3 (1.9-68.9)	4.295 (1.1-16.3)	7.2 (1.3-41.1)	2.28 (0.6-8.0)	5.8 (1.1-32.4)
Duration of Not Free Status (years)¥	Low vs. High	1.4 (0.3-5.9)	1.1 (0.0-24.1)	2.2 (0.5-9.9)	23.1 (0.2- > 1000.0)	1.1 (0.3-4.8)	0.3 (0.0-4.6)	1.7 (0.4-6.6)	3.7 (0.3-48.7)
	Med vs. High	0.6 (0.2-2.1)	0.8 (0.1-12.5)	0.6 (0.2-2.5)	22.3 (0.3- > 1000.0)	0.5 (0.1-1.7)	0.6 (0.0-11.3)	1.8 (0.5-6.6)	8.4 (0.6-111.8)
GINI§	Low vs. High	6.9 (3.1-15.3)	4.8 (1.8-12.8)	6.8 (3.1-14.8)	6.8 (2.4-20.0)	10.9 (4.7-25.2)	16.1 (5.1-51.4)	4.9 (2.3-10.5)	3.2 (1.3-7.8)
	Med vs. High	1.9 (0.9-4.0)	1.5 (0.6-3.7)	1.8 (0.9-3.8)	2.3 (0.9-5.7)	2.5 (1.2-5.3)	3.1 (1.2-8.3)	2.0 (1.0-4.1)	1.8 (0.8-4.1)

OR = odds ratio

* All adjusted for GINI, GNI/capita, total government expenditure, and % GDP spent on health except GINI (regression is adjusted for total proportion of free years, GNI/capita, total government expenditure, and % GDP spent on health).

§ Includes all states, n = 181

† Includes Free states as of 2005, n = 80

‡ Includes Partially Free states as of 2005, n = 57

¥ Includes Not Free states as of 2005, n = 44

TPFY is significantly associated with all health outcomes, except the adjusted life expectancy when TPFY goes from medium to high. When unadjusted, DFS is significantly associated with health outcomes. However, in the adjusted model, DFS is not significantly associated with any health outcome. In the adjusted model, DPFS from medium to high is statistically significantly associated with all health outcomes, but DPFS from low to high is not significantly associated with any health outcome. In the unadjusted and adjusted logistic regression, DNFS is not significantly associated with any of the health outcomes. Going from low to high the Gini coefficient is significantly associated with all health outcomes (Table 3).

Colinearity diagnosis between the intervening variables (Gini coefficient, total government expenditure, GNI/capita, and % of total GDP spent on health) revealed that all the correlation coefficients were less than 0.40 and multicolinearity issues did not confound the results. In this analysis, we did not consider the relative impact of each intervening variable on the analysis. Such analysis, for example looking specifically at the effect of increased GNI/capita on health outcomes, could provide an interesting future complement to this work.

## Discussion

Both the total proportion of free years and the duration of free, partially free, and not free status showed independent positive associations with health indicators, that remained after the adjustment for national wealth, total government expenditure, and spending on health. The total proportion of free years, which measures the cumulative level of freedom over time, showed the strongest associations with all health indicators. The Gini coefficient, a measure of income equality, was also modelled and showed a positive association with health indicators. Freedom, and relative equality in wealth may be strongly correlated in many societies, but nevertheless appear to be independently associated with improved population level health measures.

Regardless of freedom status as of 2005, low duration of freedom was associated with poorer health indicators. Furthermore, medium duration of partially free status was associated with the poorest health indicators. This suggests a detrimental link between government destabilization and health. Governmental instability because of political transition or conflict was not controlled for in this model because no such indicators currently exist.

While this study highlights some important relationships between freedom and health, several limitations should be addressed. Firstly, freedom is an oblique concept and the freedom rankings used in this paper are unavoidably problematic. Freedom House is a US-based organization with funding-ties to the US government, although other governments also contribute, and they maintain that they are independent critics of US and US allies[3]. As one may expect, their ranking system leans towards libertarian ideals of freedom and Western style democracy. However, we recognize that freedom is a multidimensional concept and that other types of freedom exist. In reality, freedom and the absence of freedom are not binary categories and any index that attempts to create a single freedom rating will be limited. However, the use of such an index is crucial to a large-scale analysis and our use of Freedom House rankings in this paper was dictated by its influence on other global ranking systems. While freedom as a political construct may vary in definition, the freedom to exercise fundamental rights as a person is recognized in international human rights law and conventions as an essential basis for human dignity. Dignity, in turn, is fundamental to human wellbeing, perhaps even more broadly than health. It may be that dignity is in the final causal pathway between freedom and health, but this cannot be ascertained with the data available to us. The link between health and economic, social, and health sector variables has also been described by Ruger and Kim[15]. In relation to human dignity, they suggest that global health inequalities should be studied in conjunction with levels of social and economic development and that global efforts to reduce health inequities should focus on the worse-off countries using a multi-dimensional approach.

Another limitation of this study is the inability to determine a temporal relationship between freedom and health. While we assume that a nation's level of freedom influences the health status of its people, the health of a people may also influence the level of freedom. For example, if a people are ravaged by illness or consumed by a struggle to garner basic necessities such as food, clean water, and shelter, they may be unable to bring about political change resulting in greater political rights and civil liberties. Although our model shows the relationship between freedom and health, the temporality of the relationship cannot be determined, making causal inferences difficult. While duration of years free is significantly associated with health outcomes, the situation likely involves interplay between the two domains rather than a simple temporal or uni-directional relationship. Another possibility is that the factors that permit the natural development of freedom, for example education, and the development of a middle class, may contribute independently to health status and thus confound the relationship between freedom and health outcomes. The data for this assertion are strongest for the education of women and girls, which has shown potent population level effects on both health and development, and may be both a cause, and an outcome, of freedom[16].

Furthermore, the patterns and timing of freedom transitions vary greatly between countries: for example, some countries have recently transitioned to freedom, to lack of freedom, or have long histories of partial freedom. While we did not attempt to classify and analyse the influences of these specific transitions, individual country histories of freedom undoubtedly play into present health outcomes. Other studies have analysed the linkages between governance and health in countries undergoing common transitions. For example, a recent analysis of post-communist countries transitioning out of communist rule shows a distinct relationship between democratisation and health indicators[17].

In contrast, this paper offers a broad overview of the relationship between historical and cumulative freedom levels and health of all countries regardless of social, political, and economic histories. The TPFY looks at cumulative freedom levels over 34 years and does not consider current freedom levels. However, in the calculation of DFS, DPFS, DNFS we classified countries according to their current (as of 2005) freedom status and measure the duration of the particular freedom level in the country.

Another consideration in the interpretation of our results is the lack of data for some countries. While we included countries with missing data in the analysis, we excluded missing data points from our calculation of freedom levels over time. Also, countries with missing data points for particular indicators were excluded from specific multivariate analysis if necessary. Not unexpectedly, missing data was more common countries with histories of partial or lack of freedom. Also, we expect there was variability in data quality between countries. While unavoidable in an analysis of this nature, these questions of data reliability are important considerations for the reader.

The fundamental mechanisms behind the association between freedom and health have not yet been identified and future research might explore the specific social, economic, and cultural components of freedom, however defined, the effect of conflict on the interplay between freedom and health, the characteristics of the temporal relationship between freedom and health and the impact of such health and freedom related factors as stress, depression, substance use and violence on the one hand, and dignity and self-efficacy on the other. However, our findings of associations between freedom and health underscores the increasingly accepted concept that human health is influenced by macro-level political forces as well as by immediate environments and personal choices[18]. This suggests that global efforts to advance political freedom and human dignity will have far ranging effects on the health of individuals and populations.

An implication of these findings is that donor investments in health are likely to have more impact on health outcomes in freer societies than in less free ones. This brings us to a fundamental challenge in health and development work globally--the disproportionate burden of poor health outcomes in the least free, least equitable (as defined here by the Gini coefficient) and poorest states. Donor aid, which attempts to address poor health outcomes and to avoid involvement in democratization is often cited as a "soft power" approach which might bypass the traditional rights-based approaches of (Western) development aid. Our findings suggest that this is a wrong-headed dichotomy: freedom and health are correlated, and health assistance will do more if the goals of expanding civil and political freedoms are not divested from giving. This may pose a special challenge to the new US administration, which in its early forays into this arena appears to be seeking new approaches toward development and eschewing what some would see as an overly moralistic focus on freedom as defined by the previous administration.

## Competing interests

The authors declare that they have no competing interests.

## Authors' contributions

SLB carried out the statistical analysis and writing of the methods and results. EMD researched and developed the database. VLD was responsible for writing the introduction and discussion, and assisted in writing the results. MRJ provided support and assistance throughout the statistical analysis. JO, CB, and KW made important content contributions and assisted in writing first drafts and redrafting the manuscript. EJM conceived the study and participated in its writing and coordination.

All authors have read and approved the final manuscript.
